# Patient-Specific Variables Determine the Extent of Cellular Senescence Biomarkers in Ovarian Tumors In Vivo

**DOI:** 10.3390/biomedicines9040330

**Published:** 2021-03-24

**Authors:** Paweł Uruski, Justyna Mikuła-Pietrasik, Eryk Naumowicz, Kamila Kaźmierczak, Andrey N. Gaiday, Jan Królak, Błażej Nowakowski, Rafał Moszyński, Andrzej Tykarski, Krzysztof Książek

**Affiliations:** 1 Department of Hypertensiology, Poznań University of Medical Sciences, 61-848 Poznań, Poland; puruski@ump.edu.pl (P.U.); jan.krolak@ump.edu.pl (J.K.); tykarski@o2.pl (A.T.); 2 Department of Pathophysiology of Ageing and Civilization Diseases, Poznań University of Medical Sciences, 61-848 Poznań, Poland; jmikula@ump.edu.pl; 3 General Surgery Ward, Medical Centre HCP, 61-485 Poznan, Poland; eryknaumowicz777@gmail.com; 4 The Greater Poland Cancer Center, Department of Surgical, Oncological, and Endoscopic Gynecology, 61-866 Poznań, Poland; kamilka35@icloud.com (K.K.); blazej@me.com (B.N.); 5 Department of Obstetrics and Gynecology, West Kazakhstan Marat Ospanov Medical University, Aktobe 030008, Kazakhstan; a.gaiday@mail.ru; 6 Division of Gynecological Surgery, Poznan University of Medical Sciences, 60-535 Poznan, Poland; rafalmoszynski@gmail.com

**Keywords:** aging, biomarkers, cellular senescence, FIGO, malignant ascites, ovarian cancer

## Abstract

The mechanisms and clinical significance of the cellular senescence of tumor cells are a matter of ongoing debate. Recently, the triggers and molecular events underlying spontaneous, replicative senescence of primary epithelial ovarian cancer cells were characterized. In this study, we reanalyzed tumors obtained from ovarian cancer patients with respect to the expression of the senescence biomarkers SA-β-Gal and γ-H2A.X and the proliferative antigen Ki67. The results showed that the tumors displayed strong heterogeneity with respect to the expression of analyzed markers. The expression of SA-β-Gal and γ-H2A.X in the oldest patients (61–85 y.o.) was significantly higher than in the younger age groups. Conversely, the area of Ki67-positive cancer cells was greater in younger individuals. At the same time, there was a positive correlation between SA-β-Gal expression and calendar age in FIGO III–IV and malignant ascites-positive patients. The γ-H2A.X positively correlated with age in the whole group, FIGO III–IV, and ascites-positive patients. Ki67 levels correlated negatively with the age of patients among those same groups. Collectively, our study indicated that organismal aging may determine the development of the senescence phenotype in ovarian tumors, particularly in patients with advanced disease and those accumulating malignant ascites.

## 1. Introduction

Cellular senescence refers to a phenomenon in which normal cells irreversibly lose their ability to proliferate, degenerate morphologically, and display upregulated expression of genes and secretion of proteins contributing to the development of an inflammation-like, procancerogenic environment [1]. Over the past two decades, considerable evidence has accumulated that cancer cells may also undergo senescence in response to clinically relevant doses of ionizing radiation and chemotherapy [2]. Apart from this therapy-induced senescence, an increasing number of reports indicate that cellular senescence may also occur spontaneously in cells that were not subjected to any therapeutic approach [3].

Ovarian cancer is the fifth leading cause of cancer-related deaths in Western countries and the leading cause of death from gynecological tumors [4]. The incidence of this disease rises progressively with age regardless of its histological subtype [5]. The same applies to cancer cell aggressiveness [6] and dissemination within the peritoneal cavity [7]. Our group recently revealed that ovarian tumors obtained from chemotherapy-naïve patients contain a significant fraction of senescent cells. Potential triggers of this phenomenon and its molecular mechanisms have been further delineated using cell cultures established from those tumors [8]. At the same time, no attempts have been made to address the role of patient aging in the development of senescence of cancer cells in tumors in vivo.

The current study was designed to verify whether there is any relationship between the calendar age of ovarian cancer patients and the development of a senescence phenotype in their tumors in vivo. To this end, two highly acknowledged biomarkers of senescence, the enzyme senescence-associated β-galactosidase (SA-β-Gal) and phosphorylated variant of histone H2A.X (γ-H2A.X) [9], along with proliferative antigen Ki67, were planimetrically quantified and then analyzed with respect to aging in the whole cohort and in patients categorized according to two clinical criteria, that is, their FIGO stage and the presence of malignant ascites.

## 2. Materials and Methods

### 2.1. Chemicals

Unless otherwise stated, all chemicals were from Merck (Darmstadt, Germany). Plastics and other consumables were from Nunc (Roskilde, Denmark).

### 2.2. Ovarian Tumors

The study was performed using tumors obtained during cytoreductive surgery from 24 women with high-grade serous ovarian cancer (HGSOC). None of the patients were treated with chemotherapy before the surgery. Detailed characteristics of the patients are presented in Table 1.

The stage of the disease was determined according to the criteria of the International Federation of Gynecology and Obstetrics. All specimens were identified as cancerous using standard H+E staining. The patients were included in the study according to subjective ultrasound assessment pointing to the presence of ovarian malignancy. The exclusion criterion was a neoadjuvant chemotherapy before the cytoreduction. The study was approved by an institutional ethics committee (consent number 578/18), and all patients gave their informed consent.

### 2.3. Histochemistry and Immunolabeling for Markers of Senescence and Proliferation

The tumors were fixed in 4% formalin for 24 h, embedded in paraffin, sectioned at 3 μm, and mounted on SuperFrost Plus adhesive microscope slides (Thermo Scientific, Waltham, MA, USA). Deparaffinization, rehydration, and epitope retrieval were conducted using Envision Flex Target Retrieval Solution (Dako, Glostrup, Denmark) at 97 °C for 50 min in a water bath. Endogenous peroxidases were blocked with Dako EnVision FLEX Peroxidase-Blocking Reagent (Dako). Then, sections were incubated in Leica Biosystems Novocastra Protein Block (Novocastra Reagents, Wetzlar, Germany) for 10 min. Histochemical detection of senescent-associated β-galactosidase (SA-β-Gal) was performed essentially as described by Dimri et al. [10] with X-gal as the substrate. As per the evaluation of the phosphorylated variant of histone H2A.X (γ-H2A.X), the specimens were incubated overnight with rabbit gamma-H2AX [p Ser139] antibody (Novus Biologicals, Centennial, CO, USA, # NB100-384) or with a monoclonal mouse anti-human Ki67 antibody (Dako, # M7240), both diluted in Dako EnVision FLEX Antibody Diluent (Dako), 1:500. Antigen visualization was performed using Envision Flex (Dako). The staining results were visualized under an Axio Vert.A1 microscope (Carl-Zeiss, Jena, Germany).

### 2.4. Planimetric Analysis

The green-stained area reflecting the presence of SA-β-Gal or brown-stained area reflecting the presence of histone γ-H2A.X and proliferative antigen Ki67 were quantified using ImageJ v1.53e software (http://rsb.info.nih.gov/ij/, accessed on 8 March 2021). Twenty × 100 randomly selected fields per tumor were examined. The results were expressed as a percentage (%), and the whole area of a specimen was treated as 100%. The fields examined did not include tumor margins to exclude any mesothelium-derived reactions.

### 2.5. Statistics

Statistical analysis was performed using GraphPad Prism^TM^ v.5.00 (GraphPad Software, San Diego, CA, USA). The means were compared with the Mann–Whitney test. The repeated ANOVA with a post hoc Newman–Keuls test was also used when appropriate. The correlations were analyzed using the Spearman test. Statistical significance was acknowledged when the *p* value was less than 0.05.

## 3. Results

### 3.1. Ovarian Tumors Display Heterogeneous Expression of Senescence Biomarkers in Vivo

**Figure 1:**
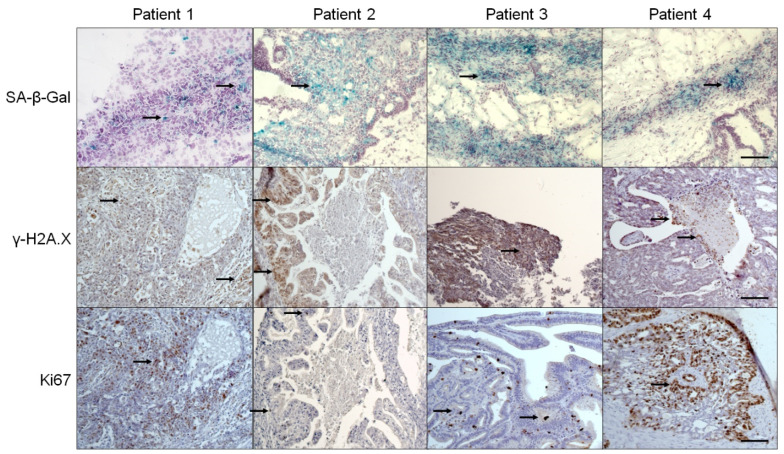
Representative staining of SA-β-Gal (green), γ-H2A.X (brown), and Ki67 (brown) showing differences in the magnitude of cellular senescence in tumors from different ovarian cancer patients. The arrows indicate exemplary positive reactions. Scale bars = 100 µm.

Tumors obtained from patients with high-grade serous ovarian cancer (HGSOC) undergoing cytoreductive surgery were subjected to analysis of cellular senescence biomarkers, that is, SA-β-Gal and γ-H2A.X, and proliferative antigen, Ki67. Planimetric analysis of specimens from 24 patients revealed that the expression of three tested parameters within cancerous tissue varied significantly between particular patients (Figure 1). The pattern of each biomarker distribution was consistent in the whole specimen, and the only tendency that could be noticed according to the comparative analysis was that the tumors expressing high levels of SA-β-Gal displayed relatively high staining of γ-H2A.X and low Ki67, and vice versa.

### 3.2. Patient-Specific Determinants of SA-β-Gal in Cancer Cells in Vivo

**Figure 2:**
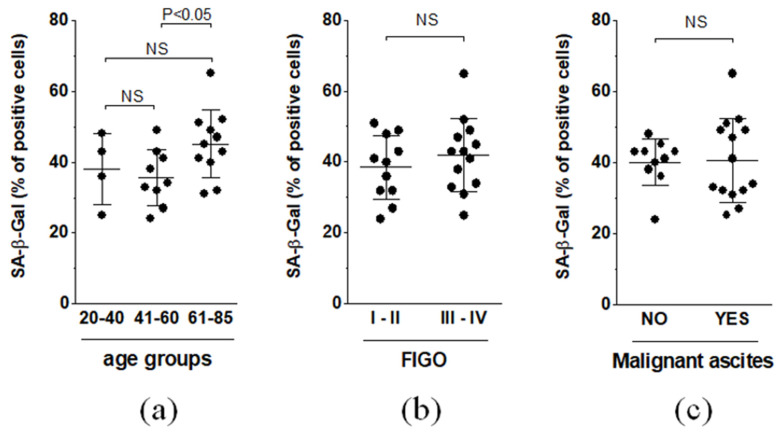
Comparison of the average areas representing the expression of SA-β-Gal with respect to patient age (**a**), FIGO stage (**b**), and malignant ascites status (**c**). Experiments were performed on 24 separate ovarian tumors obtained from different patients. The results are expressed as the means ± SDs.

To analyze the expression of the major senescence biomarker SA-β-Gal from the perspective of patient-specific variables, the enzyme expression results were grouped according to patient age, FIGO stage, and the presence of malignant ascites. Regarding the effect of calendar age, the expression of SA-β-Gal in patients 61–85 years of age was significantly higher than that in younger age groups (20–40 y.o. and 41–60 y.o.). At the same time, the average expression of the enzyme did not differ when it was analyzed with respect to the FIGO stage (I–II vs. III–IV) and the presence or absence of malignant ascites (Figure 2).

**Figure 3:**
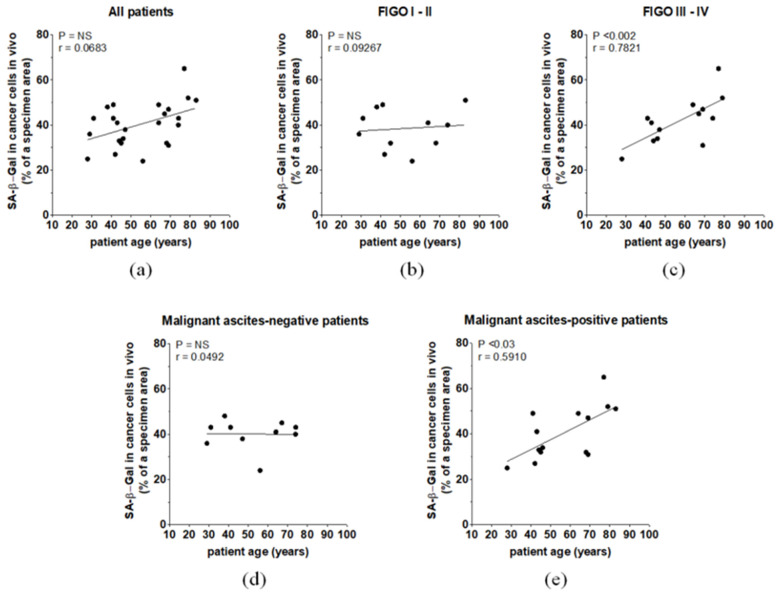
The relationships between ovarian patient age and SA-β-Gal level in the whole cohort (**a**), patients with different FIGO stages (**b**,**c**), and patients with different malignant ascites statuses (**d**,**e**).

Further correlative analyses showed that there was no correlation between the calendar age of all ovarian cancer patients and the tumor area in which the cancer cells expressed SA-β-Gal. However, when the patients were divided according to FIGO stage, a positive relationship between patient age and SA-β-Gal was observed in patients in the FIGO III–IV group and in those in whom malignant ascites accumulated in the peritoneal cavity. At the same time, patients in FIGO I–II and those without ascites did not show any correlation with SA-β-Gal staining (Figure 3).

### 3.3. Patient-Specific Determinants of γ-H2A.X in Cancer Cells In Vivo

**Figure 4:**
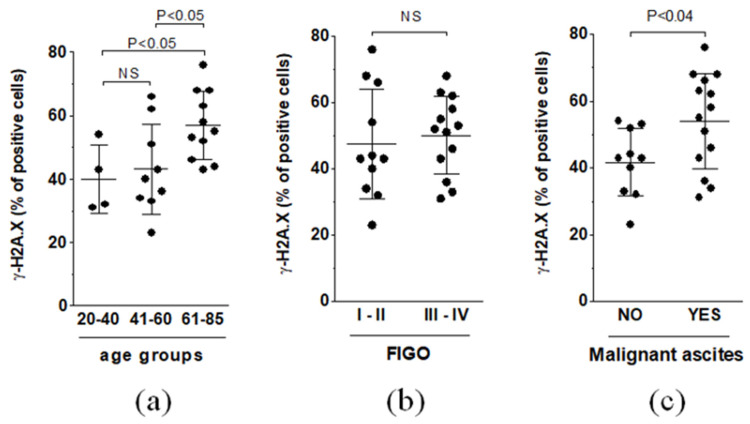
The comparison of the average areas representing the expression of γ-H2A.X with respect to patient age (**a**), FIGO stage (**b**), and malignant ascites status (**c**). Experiments were performed on 24 separate ovarian tumors obtained from different patients. The results are expressed as the means ± SDs.

A similar pattern of analysis was employed with regard to tumors characterized by the expression of the phosphorylated variant of histone H2A.X (γ-H2A.X), a marker of senescence-associated DNA damage. When three age groups were compared, tumors from the oldest patients (61–85 y.o.) exhibited a significantly higher percentage of γ-H2A.X-positive areas than the two remaining age groups. The expression of the biomarker was also higher in tumors from patients accumulating malignant ascites. At the same time, the FIGO stage did not affect the magnitude of γ-H2A.X staining in cancer cells (Figure 4).

**Figure 5:**
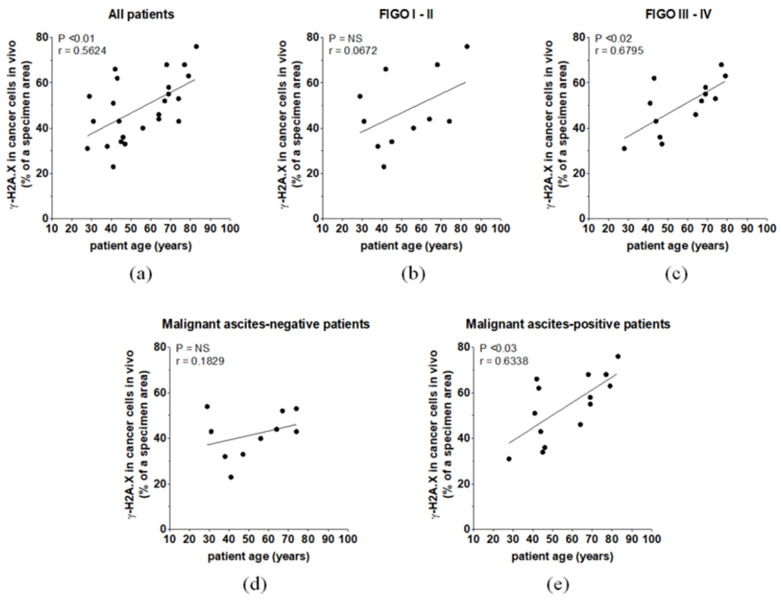
The relationships between ovarian patient age and γ-H2A.X level in the whole cohort (**a**), patients with different FIGO stages (**b**,**c**), and patients with different malignant ascites statuses (**d**,**e**).

Correlative analyses showed that the higher the calendar age of patients was, the higher the expression of γ-H2A.X in tumors was. This relationship was found for the whole group and for the patients in FIGO III–IV but not for patients in FIGO I–II. Moreover, there was a positive relationship between the extent of the γ-H2A.X-positive area and patient age in the malignant ascites-positive group. In malignant ascites-negative individuals, no such relationship was found (Figure 5).

### 3.4. Patient-Specific Determinants of Ki67 in Cancer Cells In Vivo

**Figure 6:**
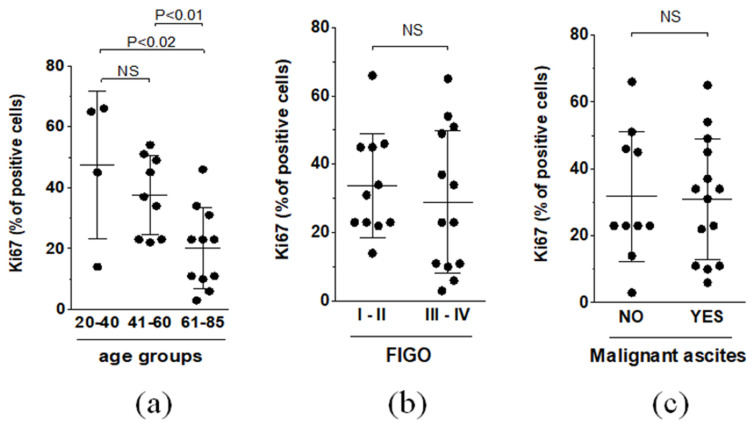
Comparison of the average areas representing the expression of Ki67 with respect to patient age (**a**), FIGO stage (**b**), and malignant ascites status (**c**). Experiments were performed on 24 separate ovarian tumors obtained from different patients. The results are expressed as the means ± SDs.

With regard to the quantification of Ki67 proliferative antigen staining, when comparing the means with respect to age, FIGO, and the presence of malignant ascites, significant differences were found for the first parameter only. Namely, the youngest patients (20–40 y.o.) and the patients representing the intermediate age (41–60 y.o.) displayed higher expression of Ki67 than the oldest patients (Figure 6).

**Figure 7:**
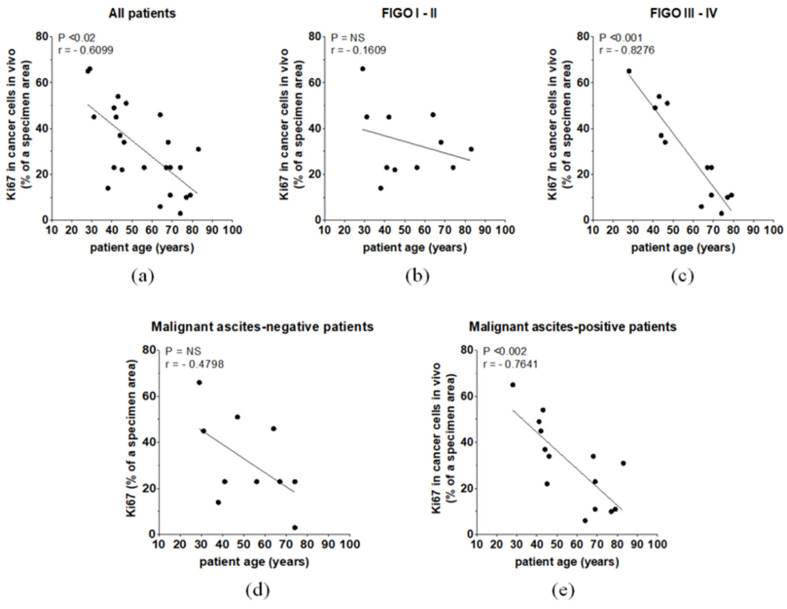
The relationships between ovarian patient age and Ki67 level in the whole cohort (**a**), patients with different FIGO stages (**b**,**c**), and patients with different malignant ascites statuses (**d**,**e**).

The abovementioned differences translated to an inverse relationship between the calendar age of patients and Ki67 staining level in their tumors. An even stronger correlation was found in the case of patients belonging to the FIGO III–IV group. In the FIGO I–II patients, no relationship between age and Ki67 was found. The magnitude of Ki67 staining in cancer cells also correlated negatively with the age of the malignant ascites-positive individuals (Figure 7).

## 4. Discussion

The clinical significance of the presence of senescent cancer cells in vivo is uncertain, as they can exert, at least theoretically, both anti- and procancerous activities. The anticancer activity of senescent cancer cells may result from their growth arrest, leading to a restriction of mitotically active cells. On the other hand, senescent cancer cells may adopt, similar to normal somatic cells, features promoting the progression of their nonsenescent counterparts, e.g., senescence-associated secretory phenotype (SASP) [11]. These critical aspects of ovarian cancer cell senescence in vivo have not yet been experimentally addressed. Before this can happen, it is worth trying to determine whether the aging observed at the cellular level (senescence) may be causally related to the aging of the patient as a whole (organismal aging), which, as emphasized in the introduction to this report, contributes to the pathogenesis of ovarian cancer.

In this study, we showed that tumors from patients with HGSOC displayed a heterogeneous distribution of the cellular senescence biomarkers SA-β-Gal and γ-H2A.X [9] and the proliferative marker Ki67. This allowed us to formulate the hypothesis that cellular senescence of cancer cells in vivo may be somehow linked with patient age. Further tests revealed that, indeed, tumors from older patients displayed a higher magnitude of senescence than lesions from younger individuals, which was previously basically found in normal tissues [12]. This observation, combined with a decreased fraction of proliferating cells in those tumors, was not always seen when the whole cohort was subjected to correlative tests. This implies that some additional factors, perhaps of clinical relevance, may matter and affect and synergize with aging. To check this scenario, we split the tested group according to FIGO stage [13] and malignant ascites status [14]. Notably, all patients categorized as FIGO I–II had tumors within the ovary or pelvis, whereas those categorized as FIGO III–IV had intraperitoneal carcinomatosis. Correlative analyses demonstrated that aging clearly correlates with tumor senescence in patients with advanced disease and those accumulating malignant fluid in the peritoneum. If any of these circumstances were not met, obtaining an unambiguous correlation for patient age was not possible.

One may theorize that the effects of ovarian cancer patient aging on tumor senescence are strengthened (or, maybe, granted?) by the specific microenvironment of the peritoneal cavity. Indeed, the omentum, which is the major location for ovarian cancer metastasis and the exact location from which the tumors are isolated, accumulates senescent mesothelial cells over time [15]. Moreover, these cells can further disperse senescence-inducing signals (so-called bystander senescence) in their neighborhood through TGF-β1 [16]. It is plausible that senescent cells whose fraction in aged people is higher [15] may also induce paracrine senescence in cancer cells in vivo. This hypothesis has support in the observations that senescent mesothelial cells lie in close proximity to ovarian cancer cells within a tumor [17] and that conditioned medium from these cells is capable of triggering the development of a senescence phenotype in primary epithelial ovarian cancer cells in vitro with GRO-1, HGF, and TGF-β1 as mediators [8]. The role of malignant ascites may be similar, as they were found to contain factors eliciting cellular senescence in normal cells [18]. The list of soluble proteins in malignant ascites that have the potential to induce oxidative stress and DNA damage and/or accelerate senescence is long and includes TGF-β1, GRO-1, IL-6, and HGF [19].

Taken together, our report shows that the presence and magnitude of senescence within tumors from ovarian cancer patients is not a random phenomenon. Instead, it is determined by patient aging, particularly when advanced FIGO stage and the presence of malignant ascites are combined. The discovery of a nonaccidental pattern of senescent cell accumulation within ovarian tumors prompts continued research on the clinical significance of these cells, and their identification should be considered an integral element of histopathological examination, with plausible diagnostic and/or predictive value. At the same time, because the classic immunohistochemistry may sometimes provide false-positive results, mainly due to incorrect pretreatment, too high concentration of a primary antibody, and some horseradish peroxidase (HRP)-associated issues [20], other methods, e.g., those based on immunofluorescence reactions, should be developed to minimize the risk of a misinterpretation.

## Figures and Tables

**Table 1:** Clinical characteristics of ovarian cancer patients included in the study.

	Number	Age [Mean (Range)]
**All patients (FIGO I-IV)**	24	54.9 (28–83)
**FIGO I-II**	11	51.9 (29–83)
**FIGO III-IV**	13	57.5 (28–77)
**Malignant ascites—YES**	14	52.1 (29–74)
**Malignant ascites—NO**	10	57.0 (28–83)

## Data Availability

The data presented in this study are available in the article.
